# Hypertensive Heart Disease: A Narrative Review Series—Part 1: Pathophysiology and Microstructural Changes

**DOI:** 10.3390/jcm12072606

**Published:** 2023-03-30

**Authors:** Valeriya Nemtsova, Annina S. Vischer, Thilo Burkard

**Affiliations:** 1 Medical Outpatient Department and Hypertension Clinic, ESH Hypertension Centre of Excellence, University Hospital Basel, 4031 Basel, Switzerland; 2 Internal Diseases and Family Medicine Department, Educational and Scientific Medical Institute, National Technical University “Kharkiv Polytechnic Institute”, 61002 Kharkiv, Ukraine; 3 Department of Cardiology, University Hospital Basel, 4031 Basel, Switzerland

**Keywords:** hypertensive heart disease, myocardial remodeling, myocardial fibrosis, vascular fibrosis, circulating biomarkers, imaging, anti-fibrotic therapies

## Abstract

Sustained hypertension causes structural, functional, and neurohumoral abnormalities in the heart, a disease commonly termed hypertensive heart disease (HHD). Modern concepts of HHD, including processes of remodeling leading to the development of various LVH patterns, HF patterns accompanied by micro- and macrovasculopathies, and heart rhythm and conduction disturbances, are missing in the available definitions, despite copious studies being devoted to the roles of myocardial and vascular fibrosis, and neurohumoral and sympathetic regulation, in HHD development and progression. No comprehensive and generally accepted universal definition and classification of HHD is available to date, implementing diagnostic criteria that incorporate all the possible changes and adaptions to the heart. The aim of this review series is to summarize the relevant literature and data, leading to a proposal of a definition and classification of HHD. This first article reviews the processes of initial myocardial remodeling, and myocardial and vascular fibrosis, occurring in HHD. We discuss important pathophysiological and microstructural changes, the different patterns of fibrosis, and the biomarkers and imaging used to detect fibrosis in HHD. Furthermore, we review the possible methods of targeting myocardial fibrosis in HHD, and highlight areas for further research.

## 1. Introduction

Cardiovascular diseases are the leading cause of morbidity and mortality worldwide. Hypertension (HTN), despite considerable progress being made in recent decades, remains a major public health problem and the most frequent modifiable risk factor associated with considerable cardiovascular morbidity and mortality. The number of people aged 30–79 years with hypertension has doubled from 1990 to 2019, despite stable global age-standardized prevalence [1].

It might seem that the lowering of the threshold for the definition of hypertension to a systolic blood pressure of ≥130 mmHg and/or diastolic blood pressure of ≥80 mm in the 2017 American College of Cardiology and American Heart Association Guidelines could explain an increase in hypertension prevalence from 18.9 to 43.5% [2,3,4]. However, in the 2018 European Guidelines, and the most recent guidelines of the International Society of Hypertension in 2020, the definition remained unchanged (≥140/≥90 mmHg) [5,6]. Regardless of the difference in definition, the comprehensive worldwide analysis of hypertension prevalence, treatment, and control for the period from 1990 to 2019 has shown a significant increase in the number of people with hypertension [1].

Numerous trials have shown that sustained hypertension causes structural, functional, and neurohumoral abnormalities of the heart, involving the ventricular and atrial myocardium as well as the epicardial and intramural coronary arteries, a disease commonly termed hypertensive heart disease (HHD). However, the published definition and classifications of HHD do not always agree, and cardiology societies and scientists have not yet come to a consensus on the terminology of these morpho-functional alterations, using terms such as “hypertensive heart disease” [7,8,9], “hypertensive cardiomyopathy” [10], and “hypertensive heart failure” [11].

The first definition of HHD as “an anatomofunctional alteration characterized by left ventricular hypertrophy (LVH) and cardiac failure in patients with systemic hypertension” was proposed in 1979 by the Criteria Committee of the New York Heart Association [7,12]. For years, definitions have continued to link HHD to LVH (Table 1). However, over time, new insights have emerged, showing that HHD encompasses a broader spectrum of hypertension-mediated organ damage beyond LVH, including cardiovascular, structural, and functional adaptations, which have impacted definitions [9]. Unfortunately, despite many studies having been performed over the years, no comprehensive and generally accepted universal definition is available.

**Figure 1:**
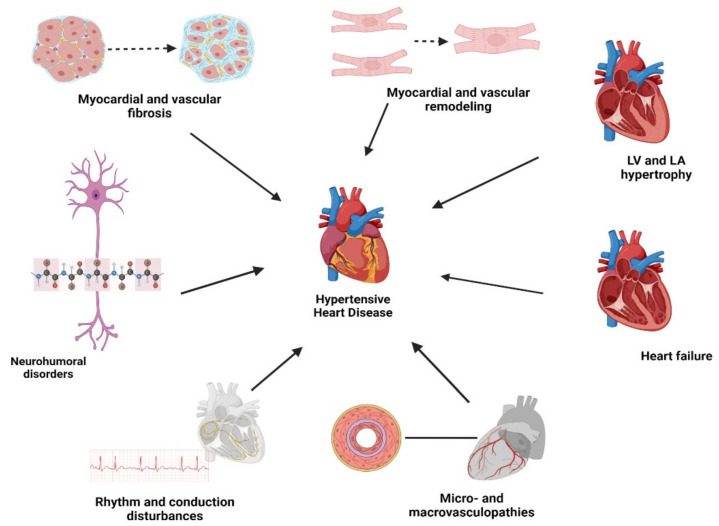
Pathomorphological, pathophysiological, and clinical patterns of hypertensive heart disease. LV – left ventricle, LA – left atrium. Figure created with BioRender.com.

Moreover, in the literature and in clinical practice, there are no generally accepted classifications of HHD that implement diagnostic criteria including all the possible changes and adaptions to the heart. Several different classifications have used the presence or absence of organic lesions, including different structures of the heart, types of LVH, and heart failure (HF) [12,26]. E. Alegría-Ezquerra et al., on behalf of the Spanish Society of Hypertension, proposed in 2006 a united classification of HHD, and presented it in such a way that it could be integrated and applied to clinical practice [12]. They used an acronym, VIA, which stands for left ventricle (V), myocardial ischemia (I), and atrial fibrillation (A). The scoring was performed according to the severity of involvement of these three categories. This is a simple and easy-to-use classification, but it has never been fully exploited over the past 12 years, and now lacks modern concepts of HHD, including processes of remodeling leading to the development of various LVH and HF patterns, accompanied by micro- and macrovasculopathies, heart rhythm, and conduction disturbances. Additionally, numerous studies have confirmed the important role of myocardial and vascular fibrosis and neurohumoral and sympathetic regulation in HHD development and progression (see Figure 1) [27,28,29,30,31].

Now, in the new universal classification and definition of HF proposed by a joint venture of international cardiologic societies (Heart Failure Society of America, Heart Failure Association of the European Society of Cardiology, and Japanese Heart Failure Society), modern concepts of neurohumoral regulation are summarized, but HF is only a part of the spectrum of HHD, which should be seen as a multi-faceted systemic disease [32]. Furthermore, over the last few years, several authors have explored the role of myocardial fibrosis and methods for its diagnosis [21,27,33]. This all grounds to analyze and summarize data from a clinical perspective.

Over the last few years, in-depth reviews of the different structural and pathophysiological processes that play a role in HHD have been published by several authors [9,21,31,34,35,36,37]. The aim of this review series is to summarize the relevant literature and to highlight the data in order to derive a definition and classification of HHD. The first part is devoted to the initial processes of myocardial remodeling and myocardial and vascular fibrosis occurring in HHD. Part 2 deals with clinical (macroscopic) aspects of HHD, including left atrial remodeling, left ventricular hypertrophy, and heart failure patterns, as well as the special features of arrhythmias and conduction disturbances related to HHD. Part 3 covers macro- and microvasculopathies, neurohumoral changes, and biomarkers, with evidence of their potential value for clinical use in HHD. Part 3 concludes with a proposal for an updated definition and classification of HHD.

To yield a clearer understanding, we have divided each review into sections focused on individual patterns or processes, but we emphasize that the manifestations of HHD are interconnected.

## 2. Methods

### Literature Search and Eligibility Criteria

A comprehensive systematic review of articles published from 1 January 2000 to 1 January 2022 was conducted using the MEDLINE (PubMed), EMBASE, Scopus, Web of Science, and Cochrane Central databases. We used the following keywords and search terms: [hypertensive heart disease], [left ventricular hypertrophy], [adverse cardiac remodeling], [cardiac remodeling], [hypertension], [hypertensive heart failure], [heart failure], [myocardial fibrosis], [hypertensive cardiopathy], [cardiac biomarkers], [circulating biomarkers], [atrial fibrillation], [arrhythmia].

We excluded studies on non-adult populations, case reports, commentaries, and abstracts. The bibliographies in the identified publications and review articles were also reviewed.

Studies were eligible for inclusion if they fulfilled the following criteria: full articles published in English in peer-reviewed journals and published from 1 January 2000 to 1 January 2022.

All authors independently evaluated the quality of the articles, searched the studies for pooled analyses based on correspondence to the study criteria, and constructed the final list of references.

## 3. Review

### 3.1. Myocardial Remodeling

Myocardial remodeling (MR) in HHD includes changes in the size, shape, and structure of the heart cavities, as well as in the biochemical, electrophysiological, and functional properties of the myocardium. It develops under the influence of a complex of various hemodynamic and non-hemodynamic factors. Experimental evidence suggests that chronically elevated blood pressure negatively impacts the normal geometry and function of both the ventricles and atria—primarily the left ventricle (LV) and left atrium (LA), which simultaneously increase in size as the initial adaptation to a deteriorating function [38].

Consequently, there is a variety of interrelated alterations between cardiomyocytes, the interstitial space, and the coronary microvasculature develop [21]. These changes determine mechanical dysfunction, thus providing the basis for cardiomyocyte malfunction, which is associated with LV hypertrophy and predisposes the LV to diastolic or systolic dysfunction [21]. As a result of the above-mentioned processes, atrial stretching develops due to long-term increased intra-atrial pressure, and this leads to atrial dilatation, dysfunction, and arrhythmia [39]. Other changes in the atrial and ventricular myocardium include electrical, neurohumoral, and structural remodeling, as well as the activation of the autonomic nervous and renin–angiotensin–aldosterone system (RAAS). Increased sympathetic nervous system activity enhances plasma renin activity, increasing the levels of angiotensin and aldosterone that stimulate cardiac hypertrophy and fibrosis [39,40]. However, the mechanisms underlying these changes due to HTN are not fully understood.

Published studies have suggested that both microvascular resistance and reactivity are impaired in hypertension, and this may further contribute to the development of both systolic and diastolic heart failure [41]. In addition, chronic pressure overload appears to lead to alterations in gene expression, resulting in both cardiac myocyte hypertrophy and alterations in the extracellular matrix, and in the later stages, this may lead to impaired relaxation and increased stiffness [41]. Polymorphisms in these genes may be partially responsible for the variability in remodeling seen in different patient populations [41].

Of note, the process of remodeling does not start at a certain blood pressure threshold in individuals with hypertension, as defined by the different guidelines, but should rather be regarded as a continuum. It is known that even prehypertensive patients are at risk for remodeling [41]. The PAMELA (Pressioni Monitorate E Loro Associazioni) study, which was performed to assess the association between prehypertension and LVH, analyzed the cross-sectional and longitudinal data of 880 untreated participants without LVH at baseline [42]. This study shows that the 10-year incidence of LVH increased progressively from individuals with normal BP levels to prehypertension and sustained hypertension (9.0%, 23.2%, and 36.5%, respectively) [42]. These data suggest that alterations in cardiac structure and function may already arise with non-optimal BP, even without overt HTN [22,42].

Additionally, these results were supported by a meta-analysis performed by Cesare Cuspidi et al. (2018) examining echocardiographic variables derived from pooled data of pre-HTN, which also clearly showed that pre-HTN may be associated with abnormalities in LV structure and function [43]. The authors demonstrated that subjects with pre-HTN exhibited mean LV mass index, relative wall thickness (RWT), LV diastolic function, and LA size values that were between those of normotensive and HTN subjects [43].

Thus, on a structural level, HHD is not just a matter of LVH, but the result of complex myocardial, cellular, and tissue arrangements, leading to changes in the shape, size, and function of the LV and other cardiac chambers.

### 3.2. Myocardial and Vascular Fibrosis

Myocardial fibrosis (MF) is considered one of the key features of hypertensive MR. Myocardial stiffness, which is thought to be a fundamental characteristic of hypertensive maladaption, is associated with fibrosis, altered contractile and relaxation properties, and changes in cardiac cellularity (especially perivascular inflammation) [44].

MF is classified into two principal types: interstitial (or reactive, diffuse) and replacement (or reparative) fibrosis [33,45]. Reparative or replacement fibrosis is a response to local signals and is characterized by small foci (focal fibrosis) of dead or apoptotic cardiomyocytes forming microscars. This type of fibrosis may occur in myocardial infarction or cardiomyocyte death due to other causes [45,46]. In contrast, myocardial reactive fibrosis (interstitial fibrosis, MIF) occurs in response to increased metabolic and hemodynamic load, and leads to the accumulation of fibrous tissue in the interstitial and perivascular space, without notable cell loss. It may occur in HF, where the myocardium endures hemodynamic overload, or in different non-ischemic heart conditions, such as age-associated changes, hypertensive heart disease, aortic valve stenosis, diabetic cardiomyopathy, and hypertrophic cardiomyopathy [33,45]. Of note, it is unclear whether these two types of MF are different processes, as they can appear simultaneously and may contribute to the structural and functional cardiac changes [46]. Moreover, to date, despite extensive research, the exact role of cardiac fibrosis remains unclear, including whether it is a causal, contributing or adaptive mechanism in the pathogenesis of heart diseases including HHD [30]. Reparative fibrosis, at least initially, is an adaptive repair response that is reversible and might potentially be necessary to maintaining the integrity of the heart [30]. Reactive cardiac fibrosis is assumed to be the result of an adaptive process that supplies structural support for heart preload. However, the fibrogenic process is a vicious cycle, leading to a further increase in arterial stiffness and fibrosis that gradually extends into the interstitial space. Experimental evidence suggests that it develops as a result of alterations leading to the predominance of collagen fiber (types I and III) formation and deposition over degradation and removal, relative to the mass of cardiomyocytes within the myocardial interstitium [21,33,47].

### 3.3. Extracellular Matrix (ECM)

The fibrotic remodeling of the heart not only affects the parenchyma, but also the extracellular matrix (ECM), which normally represents a three-dimensional elastic network of collagen fibers and non-structural proteins including cardiomyocytes, vessels, cardiac fibroblasts, and immune cells, playing an essential role in maintaining cellular and vascular integrity, cell signaling, and the regulation of cell–cell interactions [30]. This three-dimensional elastic network morphologically can be subdivided into three components: an epimysium, a perimysium, and an endomysium [44]. The epimysium is located on the endocardial and epicardial surfaces of the myocardium and provides support for endothelial and mesothelial cells. The perimysium predominantly surrounds bundles of muscle fibers. The endomysium, formed from the perimysium, surrounds individual muscle fibers and supports connections to cardiomyocyte cytoskeletal proteins across the plasma membrane. This latter is also the source of ECM scaffolding for blood vessels [44]. The ECM metabolism governs processes of collagen synthesis, processing, cross-linking, and degradation, and the extent and distribution of MF results from the imbalance between these processes [48]. The role of genetic predisposition in collagen deposition has also been discussed [49]. In 1988, the cardiac fibroblast was identified to be the major source of collagen fibers, especially type I collagen, in the heart [30]. To date, numerous studies support the significant role of cardiac fibroblasts in the initiation, modulation, and execution of ECM remodeling [30].

It was also determined that type I and type III fibrillar collagens are major components of the cardiac ECM. Type I collagen represents approximately 85% of the total collagen proteins and builds thick, highly cross-linked fibers that are important for strength, whereas type III collagen (approximately 11%) provides elasticity to the ECM by forming flexible, thin fibers [30,44]. The type I collagen to type III collagen ratio is quite stable, and can be considered as one of the most important characteristics of cardiac ECM. In the light of new data, it has become increasingly clear that not only is ECM quantity important, but ECM quality is too [30,44]. These quantitative and qualitative changes in the ECM can not only impair cardiomyocyte contractility and relaxation, but also disturb electrical conductivity, as well as regional nutrient and oxygen diffusion. Experimental data derived from hypertensive subjects and hypertensive animal models show that pressure overload leads to an excess deposition of the stiffer type I collagen in the myocardium interstitium over the more elastic type III collagen, which results in increased tissue stiffness and, as a consequence, a decreased rate of myocardial relaxation, leading to diastolic dysfunction [30]. With increasing collagen accumulation, systolic dysfunction also occurs [36,50].

### 3.4. Clinical Role of Fibrosis

A large body of evidence from animal models and human patients suggests that LV pressure overload induces interstitial and perivascular fibrosis [51]. In dog models of chronic HF, it was demonstrated that myocardial reactive fibrosis might lead to hypoxic and dysfunctional collagen-encircled cardiomyocytes [52]. Conrad and colleagues (1995) found an association of HF development with marked MF, increased passive stiffness, and impaired contractile function in aging spontaneously hypertensive rats (SHR) relative to age-matched non-hypertensive control animals [53]. The available data suggest that reactive fibrosis may precede the development of heart failure with preserved ejection fraction (HFpEF), and correlates with disease severity and worse clinical outcomes in patients with HF of various etiologies [21,30,50]. The accumulation of fibrotic tissue, and the realignment of collagen and cardiomyocytes in MF, disrupts the myocardial architecture, contributes to impaired myocardial contractility, determines mechanical, electrical, and vasomotor dysfunction, and leads to LV dysfunction, promoting the progression of HF, arrhythmias, and impaired myocardial oxygen availability, which ultimately influences the clinical course and outcome of HF patients [27,51]. The presence and extent of fibrosis have been shown to be associated with morbidity and mortality in patients with HF [33,48], and also predict the effectiveness of long-term HF therapy (e.g., beta-blockers are likely to be more effective in HF patients with less fibrosis) [33]. Additionally, the degree of collagen cross-linking (i.e., the ratio of stiff insoluble to soluble collagen) has been found to be associated with LV stiffness and diastolic dysfunction in HHD [30], and with HF hospitalization in patients with HHD and HF [21]. Recently, mounting evidence has suggested that fibrosis is more relevant in HHD than in other causes of HF [23,44]. Importantly, an early transition of cardiac fibroblasts to myofibroblasts prior to cardiac hypertrophy development is considered to be one of the major features of HHD [23].

Vascular fibrosis itself is characterized by increased intima-media thickening and vascular stiffness in small and large arteries [30,52]. In large vessels, vascular stiffness leads to hemodynamic damage to peripheral tissues. Extending from small arteries, fibrosis replaces parenchymal tissue, leading to hypertension-associated target organ damage (in the heart, kidney, and brain) [52]. Furthermore, vascular fibrosis caused by long-standing HTN could play an important role in the development and progression of structural MR, leading to a decline in LV function and HF [27,30,33,47,49].

With regard to the right ventricle, a few studies confirm the relationship of RV fibrosis with end-diastolic wall stress [30]. Using RV trabecular strips of pulmonary artery-banded rodent models, Rain et al. showed that only rats with severe RV dysfunction had RV myofibril-mediated and fibrosis-mediated stiffness and an elevated ratio of collagen I to III [54]. In general, most of our knowledge about RV fibrosis stems from research focused on the LV. The role and composition of the ECM in the RV are considered to be similar to those of the LV. In a recently published study investigating post-mortem macroscopic and microscopic differences in the RV structure between hypertensive patients and controls, there was no evidence of interstitial fibrosis [55,56]. It was not possible for the authors to answer the question of whether RV muscular remodeling is evident during hypertension [55,56]. However, older data received from autopsy samples from non-cardiac patients show that a normal RV has a higher collagen content than the LV (7.4% vs. 5.5% for RV and LV, respectively) [30]. To understand the contribution of RV reactive fibrosis to cardiac remodeling and HF in HHD, and its role as a predictor of disease progress and cardiovascular outcomes, further research is needed.

### 3.5. Value of Circulating Biomarkers for Myocardial Fibrosis

Endomyocardial biopsy is the gold standard method for diagnosing MIF, but it is invasive, not sufficiently widely available, and offers uncertain benefits in clinical practice. Therefore, attempts have been made to establish circulating biomarkers as non-invasive alternatives to detect early changes in routine clinical practice. It is postulated that inflammatory cytokines, chemokines, and reactive oxygen species may be more important in reparative fibrosis, while mechanical stress, the RAAS, and fibrogenic growth factors appear to be involved in both reparative and reactive fibrosis [33]. However, most of the markers are not cardiac-specific, and therefore, changes in their concentrations could be influenced by comorbidities affecting collagen metabolism [21,45]. To date, the clinical application of circulating biomarkers of MIF has met several limitations, as most of the proposed biomarkers have not been shown to sufficiently reflect the structural, functional, or molecular alterations associated with MR in patients with HHD, or a lack of significant association has been found with histologically proven MIF [21]. In Table 2, we present an overview of biomarkers that contribute to vascular fibrosis and ECM remodeling, with evidence from hypertensive animal models or patients with hypertension, hypertensive heart disease, or HF due to hypertension without ischemic heart disease or metabolic disorders (e.g., diabetes mellitus, obesity), along with their possible roles and influence.

The best of the studied circulating biomarkers associated with prognosis are B-type natriuretic peptide (BNP) and Troponin, which do not reflect MF, but pressure overload/wall tension and cardiomyocyte damage, respectively. To date, the most convincing association with histologically proven MIF has been demonstrated for the serum carboxy-terminal propeptide of procollagen type I (PICP), serum amino-terminal propeptide of procollagen type III (PIIINP), and serum collagen type I telopeptide (CITP) to serum matrix metalloproteinase-1 (MMP-1) - CITP:MMP-1 ratio [33,45]. Ravassa and colleagues identified a subgroup of hypertensive patients with HF with a higher independent risk of HF hospitalization and CV mortality compared to other patients in their study via the application of a combination of two circulating biomarkers—serum CITP:MMP-1 ratio and serum PICP—leading to the development of a distinct phenotype of MF [21,57].

**Table 2:** Selection of circulating biomarkers that reflect mechanisms of extracellular matrix remodeling and vascular fibrosis, and their possible roles.

Marker	Levels in Studied HHD/HTN/HHF	Role in Collagen Metabolism	Effect on Fibrosis	Study: HHD/HTN/HHF	Author, Year
Aldosterone	Increased	Profibrotic biomarker	Stimulates myocardial fibroblasts to synthesize and secrete procollagen into the ECM [58]	CHF with elevated plasma levels of natriuretic peptides, and LV EF ≤ 40% [48] HFpEF [58] HTN [52]	MR. Zile et al., 2019 [48] Paulus W.J., 2021 [58] Harvey A. et al., 2016 [52]
Angiotensin II ( Ang II)	Increased	Profibrotic biomarker	Can stimulate myofibroblasts through ANG II type 1, inhibits fibroblast apoptosis, stimulates fibroblast migration, and inducts TGF-β1. This promotes fibroblast proliferative, synthesis, and secretion activities of procollagen I and III [30,44,51]	HHD [44] HTN [52]	Schimmel K. et al., 2022 [30] Frangogiannis N.G., 2021 [51] Berk B.C. et al., 2007 [44] Harvey A. et al., 2016 [52]
Endothelin-1 (ET-1)	Increased	Profibrotic biomarker	Endothelin-1 secreted also by fibroblasts, cardiomyocytes, and macrophages and can bind to ET cardiac fibroblasts receptors, contributing to the development of myocardial fibrosis.	HTN [52,59]	Schimmel K. et al., 2022 [30] Fang T. et al., 2019 [59] Harvey A. et al., 2016 [52]
C-reactive protein (CRP)	Increased	Profibrotic biomarker	CRP directly induces collagen matrix and α-SMA expression in cardiac fibroblasts in vitro or promotes this fibrosis response in the presence of Ang II in vivo and in vitro [60]	with HFpEF [58,61] HTN [60]	Sanders-van Wijk S. et al., 2015 [61] Paulus W.J. et al., 2021 [58] Zhang R. et al., 2010 [60]
Soluble suppression of tumorigenicity-2 protein (sST2)	Increased	Profibrotic biomarker	Stimulate myocardial fibroblasts synthesize and secrete procollagen into the ECM [58]	CHF with elevated plasma levels of natriuretic peptides, and LV EF ≤ 40% [48] HFpEF [58]	MR. Zile et al., 2019 [48] Paulus W.J. et al., 2021 [58]
Interleukin 6 (IL6)	Increased	Profibrotic biomarker	The soluble IL-6 receptor in combination with IL-6 is essential in increasing collagen content regulated by isolated cardiac fibroblasts, and also plays a role in mediating a phenotypic conversion to myofibroblasts [49]	with HFpEF [58,61] HTN [59] HTN [49,58]	Sanders-van Wijk S. et al., 2015 [61] Paulus W.J. et al., 2021 [58] Fang T. et al., 2019 [59] Meléndez G.C. et al., 2010 [49]
Transforming growth factor-β1 (TGF-β1)	Increased	Profibrotic biomarker	A fibrogenic cytokine that upregulates the expression of the genes encoding fibrillar collagen type I and type III [44], stimulating both myofibroblast formation and collagen production [44]. Activation of vascular TGF-β1 increases the fibronectin, collagen, and plasminogen activator inhibitor-1 (PAI-1) synthesis that stimulates TIMP [52]	HHD [44] HTN [52]	Berk B.C. et al., 2007 [44] Harvey A. et al., 2016 [52]
Growth differentiation factor 15 (GDF-15)	Increased	Profibrotic biomarker	A member of the TGF-β cytokine superfamily, one of the factors controlling a transformation of cardiac fibroblasts to myofibroblasts [62]	HFpEF [58,61] HF [62]	Sanders-van Wijk S. et al., 2015 [61] Paulus W.J. et al., 2021 [58] Rochette L. et al., 2021 [62]
Fibroblast growth factor-21 (FGF21)	Increased	Antifibrotic biomarker	Able to reverse the myofibroblast phenotype in vitro and in vivo, indicating direct protective effects against cardiac fibrosis development [23]	HHD [23]	Ferrer-Curriu G. et al., 2019 [23]
Galectin-3 (Gal-3)	Increased	Profibrotic biomarker	Stimulates myocardial fibroblasts to synthesize and secrete procollagen into the ECM (extracellular matrix) [58] and reflects the general extent of fibrosis and the severity of HFpEF [63]	CHF with elevated plasma levels of natriuretic peptides, and LV EF ≤ 40% [48] HFpEF [58] HTN with HF [28] HTN [52]	MR. Zile et al., 2019 [48] Paulus W.J., 2021 [58] Mavrogeni S., et al., 2017 [28] Harvey A. et al., 2016 [52]
Plasminogen activator inhibitor-1 (PAI-1)	Increased	Profibrotic biomarker	Inhibits fibrinolysis, reduces plasmin generation that leads to accumulation of ECM proteins and tissue fibrosis by preventing tissue proteolytic activity and reducing collagen degradation [52]	HTN [52]	Harvey A. et al., 2016 [52]
Matrix metalloproteinase-1 (MMP-1)	Increased [45] Decreased [44]	Biomarker of collagen degradation	Related to the loss of the physiological mysial collagen scaffold and accumulation of pathologic non-mysial collagen, [45] initiates the ECM degradation process by cleaving the α-chains of type I and type II [44] collagens and type III [27] collagen	HHD with HF [45] HHD with LVH [44]	López B. et al., 2015 [45] Berk B.C. et al., 2007 [44]
Matrix metalloproteinase-2 (MMP-2)	Decreased [48,64] Increased [44]	Biomarker of collagen degradation	MMP-2 degrades basement membrane proteins, fibrillar collagen peptides, and newly synthesized type I, II, and III collagens [27,64]. Stimulation of TGF-β1 signaling; increases vascular smooth muscle cell production of collagens I, II, and III; increases fibronectin secretion that lead to collagen accumulation in the vascular wall [52]	CHF with elevated plasma levels of natriuretic peptides, and LV EF ≤ 40% [48] HTN with LVH [64] HTN [52]	MR. Zile et al., 2019 [48] Ahmed SH et al., 2006 [64] Harvey A. et al., 2016 [52]
Matrix metalloproteinase-9 (MMP-9)	Decreased [48] Increased [52,64]	Profibrotic biomarker	MMP-9 has significant effects on transforming growth factor-β and other “profibrotic” proteins and profibrotic pathways [64]. Increases in MMP-9 would be expected to increase ECM accumulation [52,64]	CHF with elevated plasma levels of natriuretic peptides, and LV EF ≤ 40% [48] HTN with LVH [64] HTN [52]	MR. Zile et al., 2019 [48] Ahmed S.H. et al., 2006 [64] Harvey A. et al., 2016 [52]
Matrix metalloproteinase-13 (MMP-13)	Decreased	Biomarker of collagen degradation	Collagenolytic enzyme. The reduction would be expected to cause reduced fibrillar collagen turnover, reduced degradation, and increased ECM accumulation [64]	HTN with LVH [64] HTN with LVH and CHF [64] HHD [44]	Ahmed S.H. et al., 2006 [64] Berk B.C. et al., 2007 [44]
Matrix metalloproteinase-1/Tissue inhibitor of matrix metalloproteinase-1 (MMP-1/TIMP-1) ratio	Increased	Reflects ECM proteolytic activity	Characterizes the balance of degradation and synthesis of ECM.	HTN with HF [19,65] HTN with LVH [44]	Drazner M.H., 2011 [19] Berk B.C. et al., 2007 [44] López B., et al., 2006 [65]
Tissue inhibitor of matrix metalloproteinase-1 (TIMP-1)	Increased	Profibrotic biomarker	Increase in TIMPs inhibits MMPs enzymatic activity, which would facilitate ECM accumulation and decreased collagen degradation [64]	CHF with elevated plasma levels of natriuretic peptides, and LV EF ≤ 40% [48] HTN with LVH and CHF [64] HHD with LVH [44]	MR. Zile et al., 2019 [48] Ahmed S.H. et al., 2006 [64] Berk B.C. et al., 2007 [44]
Serum amino-terminal propeptide of procollagen type III (PIIINP)	Increased	Profibrotic biomarker	Plasma/serum concentrations reflect collagen synthesis rate [58]	CHF with elevated plasma levels of natriuretic peptides, and LV EF ≤ 40% [48] HFpEF [58] HHD with HF [45]	MR Zile et al., 2019 [48] Paulus W.J., 2021 [58] López B. et al., 2015 [45]
Procollagen type I N-terminal propeptide (PINP)	Increased	Profibrotic biomarker	Plasma/serum concentrations reflect collagen synthesis rate [58]	CHF with elevated plasma levels of natriuretic peptides, and LV EF ≤ 40% [48] HFpEF [58]	MR. Zile et al., 2019 [48] Paulus W.J., 2021 [58]
Serum carboxy-terminal propeptide of procollagen type I (PICP)	Increased	Profibrotic biomarker	Plasma/serum concentrations reflect collagen synthesis rate [58], acting as a marker of extracellular collagen type I synthesis [66,67]	HFpEF [58] HHD with/without HF [66,67]	Paulus W.J., 2021 [58] Querejeta R. et al., 2000 [66], 2004 [67]
Serum C-terminal telopeptide of collagen type I (CITP)	Decreased	Biomarker of collagen degradation	Serum concentrations related to the intensity of the degradation of collagen type I fibrils [58,66]	HHD and HF [21] HTN [66]	Querejeta. R. et al., 2000 [66] González, A., 2018 [21]
Carboxy-terminal propeptide of procollagen type III (PIIICP)	Increased	Profibrotic biomarker	Plasma/serum concentrations reflect collagen synthesis rate [58]	HFpEF [58]	Paulus, W.J., 2021 [58]

Note: Ang II—angiotensin II, CHF—chronic heart failure, ECM—extracellular matrix, EF—ejection fraction, HFpEF—heart failure with preserved ejection fraction, HHD—hypertensive heart disease, HHF—hypertensive heart failure, HTN—hypertension, LVH—left ventricle hypertrophy, HF—heart failure, MMPs—matrix metalloproteinases, TIMPs—tissue inhibitor of matrix metalloproteinases.

All these findings suggest that monitoring plasma biomarkers of myocardial ECM remodeling might provide important prognostic information with respect to ongoing adverse LV remodeling in patients with HHD in the future, but this has to be proven prospectively.

Regarding the RV, changes in the development of MF and the activity of circulating biomarkers under pressure overload conditions, such as matrix metalloproteinases (MMPs), tissue inhibitor of matrix metalloproteinase-1 (TIMP-1), galectin-3 (Gal-3), transforming growth factor-β1 (TGF-β1), and endothelin-1 (ET-1), have been repeatedly studied and emphasized in various types of pulmonary hypertension [30,68,69]. Nonetheless, the role of fibrosis in the development of RV failure and how it affects RV function is not fully understood.

### 3.6. Value of Imaging to Detection of Myocardial Fibrosis

Considering the complexity and possible risks associated with using endomyocardial biopsy to diagnose diffuse MF in HHD in routine clinical practice, there is a need for non-invasive techniques, such as advanced non-invasive imaging methods, for MF evaluation. To quantify MF, cardiac magnetic resonance imaging (CMR), including late gadolinium enhancement (LGE), is used, but diffuse MF cannot be detected by LGE, which is a serious limitation of this technique [28]. In this regard, the development of T1 mapping, a non-invasive CMR technique, may help in providing a means of evaluating the myocardial interstitial space via the measurement of myocardial extracellular volume (ECV) [45,70]. This was demonstrated in HF patients and correlated with histological data [28]. Rodrigues et al. found the highest ECV fraction values—via T1-mapping—and the lowest systolic function in patients with eccentric LVH in comparison to other LV phenotypes in patients with HHD [71]. In addition, T1 mapping was shown to be helpful in distinguishing etiologies of LVH [36]. Both native and post-contrast T1 mapping can reliably detect and quantify MIF, but this still requires methodological standardization [72]. Advanced quantitative texture analysis (TA) of biomedical images of the myocardium performed by Cardiac Computed Tomography (CCT) can also provide additional information about fibrosis, myocyte hypertrophy and scar tissue that cannot be assessed by visual analysis. However, this approach to detecting myocardial modifications via CCT images requires further verification, including by comparison with other imaging modalities, such as echocardiography and CMR [73]. Positron emission tomography (PET) imaging allows the non-invasive quantification of myocardial perfusion, and in consequence, an indirect assessment of fibrosis, through the calculation of perfusable and non-perfusable tissues (the perfusable tissue index, PTI) [27]. In this regard, the concomitant use of these techniques with circulating biomarkers of MF could enhance the diagnostic sensitivity when phenotyping MR in HHD [21].

### 3.7. Myocardial Fibrosis as Therapeutic Target

There is currently experimental evidence that certain antihypertensive drug therapies (ACE inhibitors, Angiotensin 1 (AT1) receptor blockers, mineralocorticoid receptor inhibitors, etc.) reduce the collagen content and LV stiffness in hypertensive patients regardless of their blood pressure-lowering effect [30,33,51,74]. For example, in an SHR animal model, Susic et al. showed that eplerenone, independently of its hemodynamic effects, directly reduced fibrosis and specifically reduced collagen in the RV [44,75]. Both spironolactone and eplerenone were shown to reduce PIIINP levels [48]. In the RALES (Randomized Aldactone Evaluation Study), conducted in patients with HFrEF, spironolactone was associated with a reduction in fibrosis and collagen synthesis biomarkers in the blood [76,77]. The PARADIGM-HF study (Prospective Comparison of ARNI With ACEI to Determine Impact on Global Mortality and Morbidity in Heart Failure) showed that sacubitril/valsartan may reduce profibrotic biomarkers that reflect changes in determinants of collagen synthesis, processing, and degradation, such as Aldosterone, TIMP-1, MMP-2,MMP-9, soluble suppression of tumorigenicity-2 protein (sST2), Gal-3, Procollagen type I N-terminal propeptide (PINP), and PIIINP, in a large cohort of HFrEF patients (77% with history of hypertension) [48]. Chronic treatment with the AT1 receptor antagonist losartan was associated with reduction in collagen type I synthesis and the regression of MF in hypertensive patients [30,78], but according to Díez et al., it inhibits the synthesis of collagen type I only in severe fibrosis, and not in non-severe fibrosis [79]. However, the exact mechanism by which specific antihypertensive drugs may influence fibrosis, and the extent of this effect, are not yet clearly established.

Besides the lipid-lowering, anti-inflammatory, and cardioprotective effects, statins have been shown to be effective in attenuating cardiac fibrosis both in mouse models of HHD (rosuvastatin) and in patients with systolic HF and normal cholesterol (atorvastatin) [76,80,81]. A few studies have confirmed the effectiveness of atorvastatin in reducing plasma levels of PIIINP [76,82]. On the other hand, in a sub-study of the UNIVERSE trial (The rosuvastatin impact on ventricular remodeling cytokines and neurohormones), significant increases in collagen PINP and PIIINP plasma levels were observed in patients with chronic HF treated for 6 months with rosuvastatin [76,83]. Thus, the influence of statins on MF is still controversial.

At present, no drug primarily targeting anti-fibrotic mechanisms has been approved for the treatment of MF in cardiovascular disease, but different substances are under evaluation. The first results from the phase II clinical trial PIROUETTE (The Efficacy and Safety of Pirfenidone in Patients with Heart Failure and Preserved Left Ventricular Ejection Fraction) suggest that pirfenidone may be safe and effective in patients with HFpEF [76,84]. Additionally, studies of anti-fibrotic T-cell therapy with chimeric antigen receptor (CAR) in preclinical models have shown their potential for use in cardiovascular diseases [76].

The above-mentioned data suggest that the diffuse fibrosis seen in hypertension may be reversible. However, it is also noteworthy to mention that the degradation of ECM in a hypertrophic heart might not be benign. There is a hypothesis that the transition from compensated LVH to heart failure in HHD is associated with the degradation of ECM: diastolic heart failure (with accumulated perimysium) and systolic heart failure (degradation of endomysial and perimysial components) [30,44]. Gracham and Trafford (2007) observed in an animal model of LVH and HF that a considerable disruption of collagen within the cardiac ECM and a loss of collagen coincide with chamber dilatation and the onset of clinical symptoms during the transition from compensated asymptomatic LVH to decompensated symptomatic CHF [78]. The authors concluded that the loss of collagen from the interstitium is a critical step in the development of cardiac dilatation, and that the onset of clinical symptoms of HF may reflect the end-stage nature of the disease process. López and colleagues showed in the myocardium of hypertensive patients with systolic HF that the compromise of systolic function might be related to an imbalance in the MMPs/TIMPs ratio, resulting in the excessive degradation of mysial collagen and leading to LV dilation and reduced ejection fraction [65].

## 4. Conclusions

In summary, reactive fibrosis might function as an initial adaptive mechanism, and it is important to find out at which stage and at what quantity it becomes maladaptive. Additionally, it is important to determine if there is a point of no return for reverse remodeling. Therefore, there is a clinical need to gain a deeper pathophysiological understanding and, in particular, to further evaluate and validate the prognostic impact of circulating biomarkers and advanced imaging techniques, such that they can be used to guide the initiation of possible antifibrotic treatments, and to monitor the responses to such therapy in HHD in future trials.

## Figures and Tables

**Table 1:** Evolution of the definition of hypertensive heart disease (HHD).

Author (s)	Year	Definition
The Criteria Committee of the New York Heart Association	1979	“An anatomofunctional alteration characterized by left ventricular hypertrophy (LVH) and cardiac failure in patients with systemic hypertension” [7,12]
Frohlich E. et al.	1989, 1992	“HHD is the cardiac response to the afterload imposed on the left ventricle by the progressive increase in arterial pressure and peripheral vascular resistance as a consequence of hypertensive vascular disease” [8,13]
Joseph L. Izzo Jr. et al.	2004	“HHD is a spectrum of abnormalities that represents the accumulation of a lifetime of functional and structural adaptations to increased blood pressure load. LVH, increasing vascular and ventricular stiffness, and diastolic dysfunction are prominent intermediate features of this syndrome that operate in parallel with ischemic heart disease and ultimately cause heart failure if inadequately treated” [14]
L. Michael Prisant	2005	“HHD is the target organ response of systemic arterial hypertension. It is more than left ventricular hypertrophy or heart failure, it also includes ischemic heart disease, aortic root disease, left atrial enlargement, and arrhythmias” [15].
A. González et al.	2005	“HHD can be defined as the response of the heart to the stress imposed on the left ventricle by the progressively increasing arterial pressure and is characterized by complex changes in myocardial composition that are responsible for the structural remodeling of the myocardium” [16].
E. Alegría-Ezquerra et al.	2006	“The term “hypertensive heart disease” to encompass the complex and variable set of effects that cause the chronic increase in blood pressure in the heart of a patient with hypertension and includes the presence of anatomic or biochemical signs of left ventricular hypertrophy or ventricular dysfunction, be either diastolic or systolic, of myocardial ischemia and rhythm abnormalities” [12].
Alan H. Gradman et al.	2006	“Conceptually, HHD may be thought of as those disease manifestations that can be directly related to the cardiac anatomical changes, which accompany chronic elevation of systolic and diastolic blood pressure” [17].
I. Gonzalez-Maqueda et al.	2009	“HHD is a complex and variable syndrome that usually, but not necessarily, includes clinical manifestations derived from: left ventricular hypertrophy and dysfunction, be it diastolic or systolic; myocardial ischemia; and rhythm abnormalities, all of them caused by the effects on the heart of chronically elevated blood pressure” [18].
Mark H. Drazner	2011	“HHD is a constellation of abnormalities that includes left ventricular hypertrophy, systolic and diastolic dysfunction, and their clinical manifestations including arrhythmias and symptomatic heart failure” [19].
Javier Díez	2013	“From a pathophysiological viewpoint, HHD can be defined as the cardiomyopathy that results from the response of the myocardium to the biomechanical stress imposed on the left ventricle by the progressively increasing blood pressure. Clinically, HHD is characterized by the presence of left ventricular hypertrophy in the absence of a cause other than arterial hypertension” [20].
González A. et al.	2018	“HHD is defined by the presence of left ventricular hypertrophy or LV systolic and diastolic dysfunction and their clinical manifestations, such as arrhythmias and symptomatic heart failure, appearing in patients with hypertension” [21]
A.P. Kalogeropoulos et al.	2019	“Prolonged exposure of the heart to elevated blood pressure causes a variety of changes in the myocardial structure, coronary vasculature, and conduction system of the heart, collectively known as hypertensive heart disease” [22].
Ferrer-Curriu, G. et al.	2019	“Functional or structural heart changes, leading to diastolic dysfunction, progressive left-ventricular hypertrophy, interstitial fibrosis, systolic dysfunction and chronic heart failure in subjects with defined hypertension develop a disease commonly termed hypertensive heart disease” [23].
Chike C. Nwabuo, Ramachandran S. Vasan	2020	“HHD is characterized by micro- and macroscopic myocardial alterations, structural phenotypic adaptations, and functional changes that include cardiac fibrosis, and the remodeling of the atria and ventricles and the arterial system” [9]
Tackling, G.; Borhade, M.B.	2022	“HHD refers to a constellation of changes in the left ventricle, left atrium, and coronary arteries as a result of chronic blood pressure elevation, which increases the workload on the heart inducing structural and functional changes” [24].
Lu, Y.; Lan, T.	2022	“HHD includes left ventricular hypertrophy, systolic and diastolic dysfunction, and a broader spectrum of cardiac and vascular adaptations” [25].

## Data Availability

Not applicable.
